# Effects of disability type, prior contact, and school setting on attitudes toward peers with disabilities among Saudi female students aged 7 to 12 years

**DOI:** 10.1371/journal.pone.0291274

**Published:** 2023-09-08

**Authors:** Majed M. Alhumaid, Sarah K. Alfozan, Maryam A. Alobaid, Noha A. AlNajjar, Bashaer A. Althikr Allah, Mohamed A. Said

**Affiliations:** 1 Department of Physical Education, College of Education, King Faisal University, Al-Ahsa, Saudi Arabia; 2 Department of Special Education, College of Education, King Faisal University, Al-Ahsa, Saudi Arabia; Tallinn University: Tallinna Ulikool, ESTONIA

## Abstract

**Background:**

Inclusive educational practices enhance engagement among students with disabilities in school settings. This study aimed to investigate: (i) the general attitudes of non-disabled female Saudi Arabian students toward their peers with disabilities, with a particular focus on the general attitudes towards those with hearing disabilities (HD), intellectual disabilities (ID), and behavioral problems (BP), and (ii) the relationships between three selected student-related characteristics (type of school, in-school contact with peers with disabilities, and out-of-school contact with peers with disabilities).

**Method:**

Using a sample of 678 participants aged 7–12 years old, we tested the impact of personal and contextual factors (age, type of peer disability, type of school, in-school interaction with peers with disabilities, and out-of-school interaction with peers with disabilities on the attitudes of non-disabled Saudi Arabian elementary school students using ANCOVA linear regression analysis.

**Results:**

Regardless of the type of disability, the participants reported having positive attitudes toward peers with disabilities. The type of disability, school, and previous interactions all had a significant effect on fostering positive attitudes toward peers with disabilities, specifically, those with HD, ID, and BP. The participants had less positive attitudes towards their peers with BP compared to their attitudes towards peers students with HD or ID which were more positive and had a larger effect size. The findings also demonstrated that the participants’ attitudes toward their peers with HD or ID were influenced by their previous experience of interacting with people with disabilities as well as the type of school they attended. Participants from Saudi ARAMCO (SA) schools had more positive attitudes toward peers with disabilities compared to those from public schools, and participants from non-inclusive schools had more positive attitudes toward peers with disabilities compared to those from inclusive schools. Participants from non-inclusive schools had much more positive attitudes toward peers with disabilities than those from inclusive schools; participants who had previous out-of-school interactions with people with disabilities had significantly more positive attitudes toward peers with disabilities than those who had no previous out-of-school interactions with people with disabilities. Participants from SA schools had the most negative attitudes toward peers with BP, regardless of age.

**Conclusions:**

The findings imply that being taught in an inclusive educational setting in Saudi Arabia does not inevitably encourage non-disabled students to adopt more positive attitudes toward peers with disabilities. Therefore, with the support of their school principals, Saudi Arabian teachers working in inclusive educational settings should be encouraged to develop and implement initiatives to adopt an inclusive strategy based on group projects bringing together students with and without disabilities.

## Introduction

The term *disability* encompasses a wide range of conditions that limit basic human functional capacities. Children with disabilities often face significant challenges in their daily lives, including limited access to education, healthcare, and social opportunities. The impact on their families can be significant, affecting their daily lives and their emotional and financial well-being. Stigma, misperceptions, and negative attitudes toward people with disabilities are additional obstacles preventing those with disabilities from achieving their right to health and limiting their access to cultural and social events. McManus et al.’s [1] quantitative summary of 59 studies and 144 hypothesis tests investigating the impact of intervention programs to enhance positive attitudes among non-disabled students toward their peers with intellectual disabilities (ID) identified that children with ID are more likely to be the victims of cyberbullying and have poorer quality friendships. According to a report by Plan International, violence against minors with disabilities is also widespread [2] and such children are frequently institutionalized and excluded from wider society. Because their needs (e.g., wheelchair-accessible buildings and public transportation, sign-language interpretation, and braille) remain unaddressed and unmet, young people with disabilities face limited educational and employment opportunities and poor access to general healthcare and social services. Addressing these issues through education, awareness-raising, and policy changes is crucial to ensure that children with disabilities have equal access to the healthcare services and education they require.

Individuals and organizations concerned with the health of people with disabilities often confront moral and political dilemmas regarding how best to include and support such individuals. Such problems will intensify as societies undergo demographic shifts and an increasing number of individuals reach old age. However, the sooner a person living with a disability receives medical/rehabilitation care, the more likely it is that the negative effects of the disability and the risks of anticipated complications will be mitigated, and the better their quality of life will be. Further, the costs of preventive initiatives to achieve the above are significantly lower than the cost of managing the anticipated complications; therefore, preventive strategies designed to ensure that those with disabilities receive the appropriate medical/rehabilitative care are often hugely cost-effective compared with not doing so [3].

When children with disabilities interact with their non-disabled peers, their social skills tend to improve significantly [4]. Unfortunately, as Odom et al. [5] asserted, children with disabilities tend to lack opportunities for frequent interaction with their non-disabled peers; in part, this may be because the former are less likely to initiate social interactions with the latter. This suggests that children with disabilities are unlikely to seek to interact with non-disabled peers unless the latter are encouraged to actively initiate interactions with the former through adult intervention. To promote and facilitate positive social interactions between non-disabled children and those living with disabilities in preschool contexts, the attitudes of the former towards the latter appear crucial. Acceptance and help from peers/wider society are the most important factors determining the health and happiness of people living with disabilities [6]. As Helen Keller, writer and disabled rights activist, once observed, "The main handicap of the blind is not blindness, but the attitude of the sighted toward them." [7].

### Factors associated with children’s attitudes towards people with disabilities

According to Shiloh et al. [8], people with disabilities face a wide range of societal attitudes due to factors including culture, the nature of their disability, the personal characteristics of a particular disabled person (e.g., their gender), the characteristics of those interacting with people living with disabilities (e.g., gender, age, feelings of social awkwardness, empathy, fear of disability, personal-identity-related anxieties, and death anxiety), and the nature of the disability itself. Wang et al. [9] also affirmed that whether a non-disabled person (i) knows a person with a disability and (ii) has had contact with people living with a disability are the two most-studied characteristics related to shaping the attitudes of non-disabled people toward those living with disabilities. This study aimed to contribute to the literature in the field of inclusive education by examining the influence of certain personal and contextual factors, including age, type of disability, experience with peers with disabilities, and type of disability, on the attitudes of female elementary school students toward their peers with disabilities.

### Age

The literature on the effects of age on non-disabled children’s attitudes toward their disabled peers is inconsistent. For example, Alnahdi et al. [10] found that older non-disabled students demonstrated greater tolerance toward their disabled classmates. Armstrong et al. [11], however, found the opposite: younger non-disabled students had more positive attitudes toward their disabled classmates than their older non-disabled peers. Both Blackman [12] and Lloyd et al. [13] found that the attitudes of students without disabilities toward their peers with disabilities were unaffected by their age. Such inconsistent findings about the effect of age on non-disabled children’s attitudes toward peers with disabilities highlight the need for additional research in this area [14].

### Experience with peers with disabilities

In line with Allport’s [15] intergroup contact hypothesis, Barr and Bracchitta [16] found that people’s perceptions of members of a stereotypically maligned group tended to improve after meeting members of that group. Subsequent research on Allport’s hypothesis, however, has yielded conflicting results. Pettigrew and Tropp [17] asserted in their meta-analysis of 500 studies on inter-group contact theory that contact alone is sufficient to increase mutual understanding between communities. Seo and Chen [18] discovered, for example, that non-disabled adults who expressed more positive attitudes toward people with disabilities also reported having had more contact with the latter. Similar results were found among adolescents [19] and children [20]. Armstrong et al. [11] pointed out, however, that such interaction alone may not be enough to lessen bias among the non-disabled towards those with disabilities. They affirmed that to facilitate positive interactions and a sense of inclusivity between non-disabled children and their disabled peers, *planned experiences* must be designed and implemented. Having friends, family, or classmates with a disability or regularly interacting with people living with a disability are just two examples of how contact between non-disabled people and those with disabilities can be established through familiarity. Wang et al. [9] found that interacting with individuals with disabilities can positively affect the attitudes of the non-disabled by instigating a virtuous cycle of positive effects that lower the fears about interacting with one another that are often held by members of each group, respectively, which then tends to foster a sense of openness to the other and reduce prejudicial attitudes. Inclusive education provides opportunities for students with disabilities to learn and practice social skills, realize the importance of regular exercise in promoting physical fitness and motor skills, make friends with classmates who have a wide range of abilities, and demonstrate their progress in these areas. Nonetheless, it is important to stress that negative attitudes about those with disabilities tend to persist if demographic variables and the quality of contact were not considered [9]. To successfully foster positive attitudes toward people with disabilities in the non-disabled, the *quantity* and *quality* of contact between members of these two groups are of crucial importance. Armstrong et al.’s [11] results suggested that the importance of the *quality* of such contact exceeds the importance of the *quantity* of such contact, with positive attitudes being more related to high-quality contact (e.g., having positive interpersonal experiences) with peers with disabilities rather than having frequent contact [21].

### School-Wide inclusivity considerations

Accommodating children with disabilities in inclusive educational contexts is one of the most pressing issues confronting schools today. However, the requirements of children with disabilities in terms of providing them with quality inclusive education may not be immediately apparent to those responsible. The literature shows that many school stakeholders’ understanding of inclusion extends beyond disabilities and special education needs and studies provide several strategies for improving inclusive practices [22]. However, there are disagreements among school stakeholders about how to define inclusion; namely, between the rhetoric of inclusion found in school documents and real-life classroom practices and between the original philosophy of education and current school admission policies [23]. Therefore, to take this crucial developmental step of promoting inclusive education in mainstream school contexts, we must first understand, address and overcome the environmental, physical, and behavioral challenges that children with disabilities face in such contexts [22]. Administrative support and employee cooperation are essential for the success of any program implemented in schools; teachers must demonstrate a great deal of personal commitment, hope, and optimism in order to keep their students from lowering their expectations [24]. It is vital that current school rules and practices, as well as current cultural norms relating to children with disabilities, are evaluated [25]. Teachers’ inadvertent use of class instructions and processes that may discriminate against children with disabilities may discourage such students from participating fully; this underlines the need for enhanced training for teachers to ensure that children with disabilities are treated on a par with their non-disabled peers while ensuring that appropriate modifications are made to teaching practices to cater for their unique needs. All students must follow school rules, and punishments must be administered consistently and fairly [9]. Therefore, school staff in inclusive contexts must reconsider their approach to rule enforcement in view of the need to treat children with disabilities and their non-disabled peers with a due degree of parity. Indeed, bullying and teasing are more likely to occur when some students are held to higher standards than others. Students with disabilities will likely find it easier to reach their full potential if teachers and administrators set high expectations for all students’ behavior and academic achievement [14]. Finally, efforts to promote and celebrate diversity should be encouraged throughout the school [26]. School programs that emphasize mutual student achievement over competition, as well as those that teach students to respect and collaborate with one another, should be supported [19].

### Type of disability

The type of disability a child has influences the attitudes of those without disabilities toward them. For example, students generally reported more positive attitudes toward overt disabilities (i.e., sensory and physical) compared to less obvious ones like intellectual and learning disabilities. This effect has been observed in various samples, including instructors, students, and parents [27]. According to Wang et al. [9], students with social-emotional disorders are frequently held in negative regard by their non-disabled peers; in contrast, attitudes toward students with cognitive disabilities tend to be more positive. In terms of the positive behavioral and emotional aspects of non-disabled individuals’ attitudes towards those with disabilities, de Laat et al. [28] found that non-disabled respondents had more positive attitudes toward the deaf and hard of hearing, individuals with paralysis, and those with visual impairments than towards those with ID. de Laat et al. [28] also found that disabled individuals with paralysis and ID tend to be perceived less positively by the non-disabled than those who are deaf and hard of hearing and those living with visual impairments. Shiloh et al. [8] noted that (non-disabled) healthcare workers expressed less positive attitudes towards people with disabilities who were suffering from a diagnosed illness compared to their attitudes towards a person with a disability who had suffered an injury due to an accident. However, these differences in attitudes do not tend to manifest in social situations. Individuals with ID and BP were also less likely to be accepted than people with physical disabilities. When it comes to portraying children in general, there is evidence that children with facial deformities were treated less favorably than those with other obvious physical problems [9].

### Disability in Saudi Arabia

According to GOV.SA [29], the Kingdom’s unified national platform, 7.1% of the population is disabled. The leading cause of disability for males is car accidents, whereas for women it is severe depressive disorder. Intermarried couples have a higher incidence of disability. An increased incidence of congenital disability in children has been linked to parental handicap, advanced mother age, and insufficient prenatal care [30]. Over the past decade, the Saudi government has implemented a number of measures to guarantee the full participation in society of people with disabilities. Everyone, regardless of their health, handicap, or socioeconomic background, has free and unrestricted access to all medical options. Discrimination on the basis of any feature, including disability, is forbidden by controlled rules, which uphold the values of justice and equality. The Saudi Ministry of Education also promotes the social integration of children with disabilities by placing them in inclusive environnementals, while adhering to the culturally recognized gender segregation system [31]. "The best educational tool to achieve the social integration desired by the peoples of the world," asserts Al-Mousa [32], is inclusiveness. This was stated in a report written for the Gulf States’ Arab Bureau of Education. However, there is little, if any, study on students’ attitudes on inclusivity, particularly among females. To be more precise, "there is a lack of studies in an Islamic context that take into account children’s perceptions" [31]. Although gender segregation prevents the practice of inclusion from mirroring that seen in Western nations, it is still crucial to know whether inclusive education for children with disabilities functions in the Saudi context, not least in terms of the attitudes of pairs without disabilities. It should be emphasized also that some families still restrict their disabled relatives from attending gatherings and meetings as a form of discrimination. The family’s perspective is founded on unfavorable preconceptions about individuals with disabilities, including powerlessness, reliance, loneliness at home, poor quality of life, etc. [33].

### The present study

Although much research has investigated the determinants of the attitudes of the non-disabled toward people with disabilities [e.g. 14,26], many such variables have yet to be examined in the Saudi Arabian context and the research that has been done on this topic in Saudi Arabia is inconsistent [e.g. 34–36]. There is also a scarcity of research that attempts to establish links between individual qualities and environmental factors connected to the community, students, and school as predictors of such attitudes. Among the issues that have yet to be explored in the Saudi context are the effects of disability type on the attitudes of non-disabled elementary school girls toward their peers with disabilities. To clarify, although *overall disability* [34], *physical impairments* [37], *ID* [35], and *autism* [38] have been investigated, to date, no studies have been carried out on how having two or more disabilities affects the attitudes of non-disabled people towards those living with such circumstances. As a result, our first hypothesis (H1) is based only on the results of research conducted in international settings.

**H1.** *The attitudes of female Saudi elementary school students toward their peers with disabilities vary significantly depending on the type of disability their peers are living with*.

Saudi families are generally patrilineal and patrilocal, which means that after marriage, the Saudi wife leaves her father’s house to live with her husband. As a tribe or clan, the entire extended family lives together. Traditionally, Saudi women gather daily in the house of the eldest woman and can only leave when accompanied by a family guardian. When in the presence of a *non-mahram* (unrelated) man, some Saudi women may be fully veiled, and they are generally only allowed to socialize freely, unveiled, in the privacy of their home or school, where men are strictly forbidden to enter. In other words, the Saudi woman’s life is divided between family and school, with the exception of visiting (female) friends for a few hours with the permission of her father, her mother, or her guardian. Given these circumstances, Saudi women appear to have access to two types of social contact (i.e., whether such contact occurs in-school or is extra-curricular; the following hypothesis was developed:

**H2.** *Out-of-school contact with people with disabilities has a significant positive effect on the attitudes of female*, *non-disabled*, *Saudi elementary school students towards those with disabilities*.

Alquraini [39] looked at the current special education services and programs for Saudi students with disabilities and found that more needs to be done to provide high-quality education in the least restrictive setting possible. In particular, Saudi Arabia’s Ministry of Education could usefully consider helping teachers to improve the quality of inclusive educational programs. At the very least, the Ministry of Education should hire well-qualified teachers who have been trained in inclusive practices. Assistive technology could help students with disabilities to participate in mainstream classes and gain better access to the curriculum. The assessment process for students who are at risk of getting a poor education due to their disability should start when such children are as young as possible (e.g., preschool). This will help these students and their families to access help in securing a quality education. Alquraini [39] also said that some schools help students with disabilities in other ways, like by providing transport to school and offering psychological services and counseling. At the same time, students with disabilities often do not have access to related services like speech, physical, and occupational therapies because their schools are financially unable to hire specialists in these areas. Given the above, we present the third hypothesis:

**H3.** *The attitudes of non-disabled female Saudi elementary school students toward their peers with disabilities do not change significantly when the former have frequent in-school contact with the latter*.

In the Saudi educational system, students with mild learning disabilities attend mainstream classes with the assistance of special education services such as Special Educational Needs and Disabilities teachers. With some modifications and accommodations, these students also fully participate in activities set out in the general curriculum. On the one hand, in government-run educational institutions, students with mild to moderate cognitive disabilities are still enrolled in separate classes from mainstream students. During extracurricular activities such as lunch and recess, they spend some time with their non-disabled peers. Such students are provided with a special education program that is distinct from the general education program followed by their non-disabled peers. Students with multiple and severe disabilities are also educated in environments that prevent them from interacting with their non-disabled peers in inclusive settings [39]. Private educational institutions, on the other hand, often lack services such as occupational, physical, and speech therapists to help students to optimize their individual educational outcomes and develop their communicative, physical, and social skills. In contrast, some public institutions provide a variety of related services to students with mild to moderate disabilities, which can result in some students being taught in segregated environments, which violates their right to be taught inclusively with their non-disabled peers. Aside from government-run and private schools, Saudi ARAMCO (SA) schools (which are built and run by Saudi ARAMCO) are prevalent in its Eastern Province. The SA school system offers world-class education: it boasts spacious classrooms, small class sizes, media centers, art studios, music rooms, science laboratories, gymnasiums, sports fields, playground equipment, and the most advanced educational technology. Given these facts, the fourth hypothesis is as follows:

**H4.** *The attitudes of female Saudi elementary school students toward their peers with disabilities are substantially influenced by the type of school they attend*.

Alnahdi [35] looked at how 357 non-disabled male elementary school students in grades 3–6 felt about their peers with disabilities. He found that non-disabled students’ ages had a statistically significant effect on their attitudes toward their peers with disabilities: Older students reported having more positive attitudes toward their peers with disabilities compared to younger non-disabled students. Given this, a sixth hypothesis was formulated to investigate the effect of participant age on their attitudes towards and perspectives on peers living with disabilities:

**H5.** *Female Saudi elementary school students’ attitudes toward peers with disabilities are positively and significantly determined by their age*.

## Materials and methods

### Study design and setting

Sampling, participant recruitment, and data entry were carried out during the first semester of the 2022–2023 school year, from October 1 to December 31, 2022. A letter of invitation was sent to the principals of female elementary schools in the Eastern Province of Saudi Arabia via the Directorate General of Education. Once a principal consented to their student’s participation, the researchers went to the school to deliver invitation letters and parental consent forms to teachers for distribution to the parents of students without disabilities. This letter outlined the study’s goals, stressed the importance of protecting participants’ privacy, and assured participants that their comments would be kept confidential and used solely for this research study. In addition, teachers were instructed on how to assist participants in completing the questionnaire and how the experiment would be conducted. After receiving signed parental consent documents, the questionnaire was distributed, and responses were collected. Children who required additional explanation or had trouble comprehending the questions were helped by a member of the research team; if the issue persisted, the questionnaire was completed in the form of a face-to-face interview in a private classroom provided by the school.

The questionnaire featured two sections. The first featured a series of question items designed to gather demographic data (*age*, *type of school*) and the incidence of prior interactions with individuals with disabilities. These interactions were classified as either *in-school* (*Yes = 1; No = 0*) or *out-of-school* (*Yes = 1; No = 0*). Any participant who claimed to have interacted with a classmate or someone with a disability at her school was deemed to have had *in-school contact*; however, if a participant claimed to have a family member or friend with a disability, or played outside of school with a disabled person, they were deemed to have had *out-of-school contact*. Second, the participants were presented with three hypothetical scenarios that prompted them to make decisions regarding: (i) including a hypothetical child with HD (i.e., a child who has a severe hearing disability), (ii) including a hypothetical child with ID (i.e., a child with Down Syndrome and delayed development), and (iii) including a hypothetical child with BP. The responses to these three hypothetical scenarios served as indicators of their attitudes toward persons with disabilities in general and toward each type of hypothetical disability, respectively. Drawings and paper dolls were used to evoke responses from children.

### Participants

Participants were recruited by geographic area, then randomly selected from elementary school pupils aged 7 to 12 years. Using data from Krejcie and Morgan’s table [40], 384 students should be selected from each school type (public, private, and SA schools). As a minimum, our study required 1,275 students, allowing for a 20% attrition rate [41]. Eight all-female schools were randomly selected for each school type, with two schools in each of the four geographical zones (east, west, north, and south). Assuming a class size of 10–20 students, 48 classes were randomly selected from each school type. Participants were invited to take part in the study after eliminating those who met one or more exclusion criteria, namely (1) the presence of a disability as defined by the Saudi Ministry of Health, and (2) incomplete or incorrect questionnaires. A written informed consent form was also sent to parents via school principals prior to the start of the survey, and the absence of their signature was also considered an exclusion criterion. A total of 700 parents gave their consent for their child to participate; 22 children were withdrawn due to incomplete responses. The final sample comprised 678 female elementary school students aged 9.91±1.68 years. It should be noted that our sample consisted entirely of female students due to the scarcity, if not complete absence, of research on Saudi females in this field. This decision can also be attributed to the strict gender segregation practiced in Saudi Arabia for religious reasons. This segregation makes it challenging to conduct tests simultaneously on both girls and boys. As males are not allowed in female schools and vice versa, conducting a study with both male and female participants requires two separate research teams (one for boys and one for girls) and double the effort, particularly on administrative tasks such as obtaining permits. Additionally, it is worth mentioning that the Saudi government officially sanctioned a PE program solely for female students during the 2017–2018 school year. While PE programs have since been introduced in female schools, their implementation is not yet widespread. In contrast, PE is mandatory for male students starting from Grade 1, which might slightly distort the comparison between girls and boys. Ethical approval was obtained from the Research Ethics Committee at King Faisal University (KFU-REC-2022-AUG–ETHICS149).

### Research method

#### Questionnaire

The questionnaire participants were asked to complete was adapted from Nota and Soresi [42] with minor changes––it was designed to measure elementary school students’ attitudes toward their peers with disabilities. The questionnaire described three fictitious individuals with disabilities: one with HD, one with ID, and one with BP (aggression and temper tantrum issues). To ensure that the presentation of these hypothetical disabilities did not bias the results, simple, clear, and identical descriptions of the strengths and weaknesses of the three respective disabilities were provided to all participants; participants who needed help understanding the above all received standardized explanations to avoid the risk of undue bias [14].

The participants were asked nine questions about each of the three hypothetical individuals living with their respective disability (HD, ID, BP), including whether the participant would wish each of the three hypothetical individuals: (i) to be best friends, (ii) to play at their house, (iii) to sleep over at their house, (iv) to attend their birthday party, (v) to keep them company during recess, (vi) to be their neighbor, (vii) to talk to them, and (viii) to be their classmate. To assess participants’ attitudes toward including these three hypothetical individuals with their respective disabilities in PE class activities, the statement *I would like to play with this child during physical education class* was added. Before data collection, the questionnaire was translated from its original English version to Arabic using the procedures recommended by Banville et al. [43]. Two separate bilingual translators from the researchers’ university translated and back-translated the questionnaire. After the retranslated version was revised, five experts in psychology, physical education, and special education looked over the validity of its content. Based on the feedback, the name of the hypothetical student with HD was changed from *Carlo* to the more common Arabic name, *Sara*. The name of the student with ID was changed from *Paolo* to *Noura*; the name of the student with BP was changed from *Giorgio* to *Asma*; giving the three hypothetical students Arabic names was done to ensure that the participants would better relate to them as potential classmates. Other changes included, for example, modifying the statement *I would invite this child to my birthday party* to *I would invite this girl to my graduation party* as most Muslims do not celebrate birthdays. *I would invite this child to sleep over* was changed to *I would invite this girl to play with me after school (at a playground or in a park)* because Saudi girls are not allowed to spend the night at a friend’s house. The final modified Arabic-language version of the questionnaire featured three series of ten questions that asked about each of the three hypothetical girls with disabilities respectively, giving a total of 30 items. All responses were scored on a five-point Likert scale (*strongly disagree = 1; disagree = 2; neutral = 3; agree = 4; strongly agree = 5*). A mean score for each participant’s overall attitudes and perceptions of the three hypothetical individuals with each type of disability (HD, ID, BP) was calculated by adding all corresponding item scores and dividing by the total number of items. Higher participant scores indicated more favorable attitudes toward peers with disabilities.

#### Reliability and validity of the scale

According to Heale and Twycross [44], validity is the degree to which a concept is accurately measured using a particular research instrument; reliability is the degree to which an instrument demonstrates measurement consistency [45]. To ensure the reliability and validity of the questionnaire used to collect data for our study, the survey was pilot tested on 90 participants from three additional elementary schools (one public, one private, and one SA school). Principal component analysis and exploratory factor analysis were used to assess the construct validity of the Arabic version of the questionnaire: The Kaiser-Meyer-Olkin (KMO) sample adequacy measure was 95%, and the Bartlett test sphericity score was statistically significant (p < 0.001), indicating that the commonalities were greater than 30%. Exploratory factor analysis of the questionnaire items scores determined that the 30 items comprised a three-dimensional construct, with three components accounting for 74.144% of the total variation. The questionnaire’s reliability was calculated using Cronbach’s alpha. An overall value of 0.962 and individual values of 0.944, 0.962, and 0.976 for HD, ID, and BP, respectively, indicate high levels of instrument reliability. Di Maggio et al. [14] obtained similarly high values of 0.87, 0.90, and 0.91. Furthermore, all items included in the questionnaire are significantly correlated with their related subscales, indicating that all items achieved the measurement objective. Thus, all items in the Arabic version of the questionnaire were retained for use in the main study; the Arabic translation proved to be a valid and trustworthy instrument for measuring the perceptions and attitudes of non-disabled Saudi elementary school students toward their peers with disabilities.

#### Data analysis

Indices of normality (e.g., skew, kurtosis) were investigated via the continuous variables included in the study (Scores relative to attitudes toward disabilities in general and HD, ID, and BP in particular). Outliers were removed, the equality of variance was tested using Leven’s test, the multicollinearity problem was studied using variance inflation factors (VIFs), the linear correlations between attitudes and age were investigated using scatterplots, and correlation analysis among study variables was conducted. All dependent variables failed the homogeneity of variance and covariance assumption. The behavioural problems also failed to satisfy the equality of error variance condition. The attitudes of non-disabled participants toward students with behavioural problems were examined using a one-way ANOVA to determine whether there were statistically significant differences with the other hypothetical disabilities and according to school categories. The Student’s t-test was also used to compare attitudes toward students with behavioural issues based on the nature of previous contact. Using repeated ANCOVA testing, we also investigated the impact of school type and contacts made in-school and out-of-school on the age-adjusted attitudes of the non-disabled participants towards their peers in general, and towards those with HD or ID separately. Multiple comparisons were performed by utilizing Bonferroni’s posthoc test, effect sizes were evaluated utilizing partial eta squared or Cohen’s d, and p-values were set to 0.05 for statistical analysis utilizing IBM SPSS V.26 (Armonk, New York, United States).

## Results

### Demographics

A total of 678 girls from public, private, and SA elementary schools in the Eastern Province of Saudi Arabia completed all phases of the study. Overall, 28.91% of the participants were from public schools, 29.20% were from private schools, and 41.89% were from SA schools. The sample included 83 students aged seven, 86 students aged eight, 97 students aged nine, 94 students aged ten, 181 students aged eleven, and 137 students aged twelve. Of these participants, 95 reported having a family member with a disability while 583 did not; 80 had a friend with a disability compared to 598 who had never had a friend with a disability; 77 had a classmate with a disability compared to 601 who belonged to non-inclusive classes (a class without any students with a disability); 209 had played with a person with a disability compared to 469 who had never done so. In comparison to the 504 individuals who either had no contact or had only a single incidence of contact with a peer with a disability, 337 participants had at least one in-school contact with a peer with a disability, 269 had at least one out-of-school contact with a peer with a disability, and only 174 participants had at least one in-school and one out-of-school contact with a peer with a disability (see Table 1 below).

**Table 1:** Attitudes of Saudi girls toward peers with hearing impairment, intellectual disability, or behavioral disorders according to age, type of school, and contacts with people with disabilities.

	N	Hearing disability	Intellectual disability	Behavioral problems	Overall Attitudes
Age (years)					
7	83	4.006±0.746	3.915±0.779	3.065±0.885	3.662±0.634
8	86	4.263±0.670	4.175±0.721	3.129±0.953	3.855±0.605
9	97	4.271±0.728	4.163±0.845	3.430±1.109	3.955±0.689
10	94	4.063±0.707	3.918±0.720	3.401±0.905	3.794±0.678
11	181	4.044±0.635	3.969±0.757	3.424±0.925	3.812±0.635
12	137	3.866±0.700	3.840±0.731	3.510±0.857	3.739±0.673
Type of school					
Public	196	4.003±0.739	3.939±0.762	3.536±0.912	3.826±0.690
SAS	284	4.138±0.683	4.104±0.769	3.156±0.995	3.799±0.638
Private	198	4.026±0.686	3.854±0.740	3.471±0.853	3.783±0.649
In-school and out-of-school contacts (only)					
Yes	174	3.998±0.652	3.881±0.743	3.394±0.933	3.757±0.676
No	504	4.090±0.718	4.019±0.770	3.345±0.951	3.818±0.649
In-school contacts (only)					
Yes	337	4.017±0.697	3.881±0.784	3.341±0.913	3.746±0.666
No	341	4.115±0.705	4.084±0.733	3.374±0.978	3.858±0.642
Out-of-school contacts (only)					
Yes	269	4.088±0.651	3.974±0.738	3.463±0.903	3.842±0.650
No	409	4.052±0.734	3.989±0.783	3.288±0.968	3.777±0.659

Note: Results are expressed as Mean ± Standard Deviation; SAS, Saudi ARAMCO School.

### Associations between attitudes toward peers with disabilities with the type of disability

To examine the participants’ attitudes toward peers with disabilities in general and toward each hypothetical individual with ID, HD, and BP, the participants were asked to rate their level of agreement (*strongly disagree = 1; strongly agree = 5*) with the statements included in the questionnaire. Table 2 shows that overall, the participants agreed with all items (3.4 < score < 4.2), with 3.4 being the cut-off mean for *agree* and 4.2 being the cut-off mean for *strongly agree*, thus indicating that all participants had positive attitudes toward their peers with disabilities. Participants strongly agreed with items 4 and 8 (*I would invite this child to my graduation party* and *I would talk to this child*, respectively) and agreed with the remaining items pertaining to peers with HD. They also agreed to all items regarding peers with ID, thus indicating that all participants had positive attitudes toward their peers with ID. However, in response to the items related to the hypothetical individual with BP, they only agreed *to talk to peers with BP*, *to play with peers with BP in PE classes*, *to invite peers with BP to their graduation parties*, and *to be their house-neighbours*.

**Table 2:** Comparison of attitudes of Saudi girls toward peers with disabilities according to disability type.

	Hearing Disability	Rank	Intellectual Disability	Rank	Behavioral Problems	Rank	Overall Attitudes	Rank
1. I would like to have this child as my best friend	4.04±0.905 ^BP^	5	3.95±0.938 ^BP^	7	3.10±1.189	10	3.69±1.103	8
2. I would go play at this child’s house	3.94±0.974 ^BP^	9	3.86±1.003 ^BP^	9	3.18±1.167	9	3.66±1.106	10
3. I would invite this child to play with me after school	4.00±0.993 ^BP^	8	4.01±0.974 ^BP^	4	3.31±1.186	7	3.77±1.104	7
4. I would invite this child to my graduation party	4.28±0.847 ^BP^	1	4.17±0.913 ^BP^	1	3.48±1.175	3	3.98±1.050	2
5. I would keep this child company during recess	4.03±0.893 ^BP^	6	3.93±0.935 ^BP^	8	3.36±1.144	5	3.77±1.039	6
6. I would have fun with this child	4.00±0.920 ^BP^	7	4.00±0.939 ^BP^	5	3.33±1.092	6	3.78±1.036	5
7. I would like this child to be my neighbor	4.08±0.909 ^BP^	4	3.99±0.968 ^BP^	6	3.43±1.143	4	3.83±1.050	4
8. I would talk to this child	4.23±0.835 ^BP^	2	4.14±0.890 ^BP^	2	3.61±1.117	1	3.99±0.993	1
9. I would like to have this child as my deskmate	3.93±1.009 ^BP^	10	3.81±1.108 ^BP^	10	3.20±1.226	8	3.65±1.162	9
10. I would like to play with this child in physical education classes	4.13±0.923 ^BP^	3	4.10±0.959 ^BP^	3	3.59±1.189	2	3.94±1.060	3
Overall mean value	4.067±0.702 ^BP^		3.983±0.765 ^BP^		3.358±0.946		3.802±0.870	

Note: Results are expressed as Mean ± Standard Deviation; ^BP^ differs significantly from the group of girls with behavioral problems.

Next, by considering the participants’ ages, we compared their attitudes toward peers with disabilities of different types (HD = 1, ID = 2, and BP = 3) to see if there were any associations between age and such attitudes (see Table 3). As the equality of error variance assumption was not met, we opted for the Multiple Linear Regression test rather than GLM Univariate test. The participants’ attitudes toward people with disabilities varied considerably based on the nature of the disability (R^2^ = 0.111; *β* = -0.354; *p* = 0.001), with no effect being found that was related to the particular age of the participants. When compared to students with HD or ID, participants’ attitudes toward students with BP were significantly less positive (F = 154.66; *p* < 0.001 for both; partial eta squared = 0.40).

**Table 3:** Multiple linear regression analysis predicting the association between attitudes towards peers with disabilities and disability type.

Model		Unstandardized Coefficients		t	Sig.		95.0% Confidence Interval for B		
		B	Std. Error			R Square	Lower Bound	Upper Bound	VIF
1	(Constant)	3.826	0.115	33.126	<0.0001	0.000	3.599	4.052	
	Age	-0.002	0.011	-0.207	0.836		-0.025	0.020	1.000
2	(Constant)	4.534	0.118	38.518	0.000	0.111	4.304	4.765	
	Age	-0.002	0.011	-0.219	0.827		-0.024	0.019	1.000
	Type of disability	-0.354	0.022	-15.890	0.000		-0.398	-0.311	1.000

### Attitudes towards the three hypothetical disabilities

We tested for significant main and interaction effects of school type (public school = 1, SA school = 2, and private school = 3) on participants’ attitudes towards peers with disabilities and the effect of having had prior contact with people with disabilities (in-school contact: yes = 1; no = 0; out-of-school contact: yes = 1; no = 0) on the children’s attitudes toward the three hypothetical peers with HD, ID, or BP while controlling for the effects of participant age (see Table 4). The results of the ANCOVA tests indicated that in-school contact (F = 4.706, *p* = 0.03) and the type of school (F = 4.307, *p* = 0.014) significantly affected students’ attitudes toward their peers with HD, with a significant effect of participant age (*p* = 0.041). More positive attitudes towards peers with HD were also found among participants from SA schools compared to those attending public schools (*p* = 0.023), with the estimated mean values for attitudes towards peers with HD being 3.95±0.074, 4.177±0.045, and 4.024±0.064 for students attending public, SA, and private schools, respectively. Those attending non-inclusive schools had significantly better attitudes toward peers with HD than those attending inclusive schools (4.126±0.049 versus 3.971±0.052, *p* = 0.03).

**Table 4:** Analysis of covariance predicting age-adjusted effects of school type and contact type on Saudi girls’ attitudes toward peers with hearing or intellectual disabilities.

	Tests of Between-Subjects Effects						
	Source	Type III Sum of Squares	df	Mean Square	F	Sig.	Partial Eta Squared
Hearing disability	Corrected Model	13.808	12	1.151	2.392	0.005	0.041
	Intercept	289.762	1	289.762	602.295	0.001	0.475
	Age	2.020	1	2.020	4.198	0.041	0.006
	Type of school	4.145	2	2.072	4.307	0.014	0.013
	In-school contact	2.264	1	2.264	4.706	0.030	0.007
Intellectual disability	Corrected Model	19.983	12	1.665	2.943	0.001	0.050
	Intercept	254.085	1	254.085	448.994	0.001	0.403
	Age	0.522	1	0.522	0.922	0.337	0.001
	Type of school	8.283	2	4.142	7.319	0.001	0.022
	In-school contact	6.180	1	6.180	10.921	0.001	0.016
Overall Attitudes	Corrected Model	7.304	12	0.609	1.425	0.149	0.025
	Intercept	207.277	1	207.277	485.114	0.001	0.422
	Age	0.075	1	0.075	0.175	0.676	0.000
	Out-of-school contact	1.678	1	1.678	3.926	0.048	0.006
	In-school contact	3.016	1	3.016	7.058	0.008	0.011

Similar findings emerged regarding participants’ attitudes toward students with ID, although there was no significant age effect this time (see Table 4). Students at SA schools had more positive attitudes towards peers with ID than those at public (*p* = 0.003) and private (*p* = 0.017) schools, with mean values of 3.818±0.08, 4.133±0.049, and 3.898±0.069, respectively. There was also a significant difference between attitudes toward peers with ID among participants attending inclusive and non-inclusive schools (3.821±0.057 vs. 4.078±0.054; *p* = 0.001).

Attitudes toward students with disabilities, in general, were also significantly influenced by prior contact with persons with disabilities (out-of-school contact: F = 3.926; *p* = 0.048; in-school contact: F = 7.058; *p* = 0.008), with no effects found for participant age. Students attending non-inclusive schools had significantly more positive attitudes towards peers with disabilities compared to those attending inclusive schools (3.8980.047 vs. 3.7180.049); those with out-of-school contact had significantly better attitudes than those without (3.7410.049 vs. 3.8750.047). All effect sizes were modest when analysed using an age-based cut-off of 9.91 years for all mean estimates (see Table 4).

Since the equality of error variance assumption was not met, we opted for the Multiple Linear Regression test rather than GLM Univariate to examine students’ attitudes toward the hypothetical peer living with BP. The results indicated a significant negative correlation between holding positive attitudes toward those with BP and school type (R^2^ = 0.023; t = -2.981; *p* = 0.003). Students from SA schools exhibited the most negative attitudes toward peers with BP (*p* = 0.001; effect size f = 0.19) based on ANOVA results. The age of the participants had no effect on their attitudes toward peers with BP (Table 5).

**Table 5:** Multiple linear regression analysis predicting the association between attitudes toward peers with behavior problems with school type and prior contact with people with disabilities.

Model		Unstandardized Coefficients		t	Sig.		95.0% Confidence Interval for B		
		B	Std. Error			R Square	Lower Bound	Upper Bound	VIF
1	(Constant)	4.372	0.175	24.932	0.001	0.007	4.027	4.716	
	Age	-0.039	0.017	-2.247	0.025		-0.073	-0.005	1.000
2	(Constant)	4.360	0.207	21.049	0.000	0.023	3.953	4.767	
	Age	-.031	0.018	-1.750	0.081		-0.067	0.004	1.074
	Type of school	0.010	0.045	0.233	0.816		-0.078	0.099	1.383
	Out-of-school contact	0.043	0.062	0.698	0.485		-0.078	0.164	1.068
	In-school contact	-0.207	0.070	-2.981	0.003		-0.344	-0.071	1.427

## Discussion

The present study aimed to examine the attitudes of Saudi females elementary school students towards their peers with disabilities, including those living with HD, ID, or BP. This study also sought to determine the age-adjusted influence of type of disability, previous contact with people with disabilities and type of school on Saudi elementary schoolgirls’ attitudes towards their peers with disabilities. The findings demonstrated that, in general, participants expressed positive attitudes towards their peers with disabilities, with an overall mean score of 3.802 and a standard deviation of 0.87 derived from the five-point Likert scale used in the questionnaire. This result concurs with Alnahdi’s [35] finding that 357 male elementary school students (grades 3–6) from Riyadh, Saudi Arabia, also reported having positive attitudes toward peers with disabilities (overall mean: 24.5 from the highest-possible mean score of 40). Also, the results of the current study agree with those of Alhumaid [34] who found that most of the 972 students he sampled (aged 9–12 from both public and private all-male and all-female elementary schools in Eastern Saudi Arabia) expressed positive attitudes toward children with disabilities. Further, recent work by Szumski et al. [46] suggests that most non-disabled students regard their peers with disabilities either favourably or neutrally; however, there is a world of difference between regarding peers with disabilities favourably *or* neutrally [47]. In a meta-analysis spanning research conducted between 1990–2000, Nowicki and Sandieson [48] found that, in general, children’s attitudes toward people with disabilities require improvement, and that youngsters without disabilities preferred target peers without disabilities compared to targets with physical disabilities or ID. Nowicki and Sandieson [48] also confirmed that non-disabled females tended to be more accepting of disabled people than males, but only if (a) all individuals with disabilities were of the same gender as the participants or (b) all individuals with disabilities (male and female) were introduced to all survey participants. Furthermore, McDougall et al. [19] discovered that while the majority of ninth graders had neutral-to-favourable attitudes toward students with disabilities, over 20% had negative attitudes toward such individuals. They also discovered that the participants’ attitudes towards students with disabilities differed according to demographic factors, disability literacy levels, and contextual factors. We now examine the findings in relation to each of the above hypotheses.

### Hypothesis 1

The findings supported H1 (*The attitudes of female Saudi elementary school students toward their peers with disabilities vary significantly depending on the type of disability their peers are living with*). For each 1-unit increase in the type of disability (HD = 1, ID = 2, and BP = 3), the mean average positive attitudes score decreased by nearly 0.354. In general, participants had less favorable attitudes towards the hypothetical individual with BP; attitudes towards the hypothetical individuals with HD and ID were the most positive, respectively. Numerous studies on children [48], teenagers [49], and adults [50] corroborate these findings. Therefore, this finding concurs with the existing literature that non-disabled people’s attitudes tend to be more negative toward those with BP compared to HD and ID. According to Freer’s [26] systematic analysis of current research (2012–2019) on student attitudes toward disability, non-disabled students’ attitudes tend to be more positive toward those with more visible disabilities (such as sensory like HD and physical disabilities) than those with (often more difficult-to-manage) disabilities such as BP and cerebral and learning disabilities. Disabilities that affect an individual’s ability to communicate and interact socially tend to elicit more unfavourable attitudes among the non-disabled [16]. According to research conducted by Nowicki and Sandieson [48], younger children tend to express more positive attitudes toward their peers with physical disabilities than those living with ID. Brown et al. [49] found that high-school students were more open to participating in extracurricular activities with classmates living with physical disabilities than those who had ID. Furthermore, Barr and Bracchitta [16] verified that people without disabilities do not regard all disabilities equally and that exposure to individuals with different types of disabilities may have varying effects on the formers’ attitudes towards the latter. Understanding the extent of such disability-related bias is likely to require a multifaceted, mixed-methods approach to investigate how interactions between the non-disabled and those with disabilities affect the attitudes of the former towards the latter [51]. Since the motivations of the participants with respect to their reasons for their attitudes towards the three hypothetical individuals fell outside the scope of the present study, we can only speculate as to the reasons for these less favorable attitudes toward the hypothetical individual living with BP. Research on individuals with psychiatric disorders provides one possible explanation for these more negative attitudes toward those with BP. The more unpredictable and dangerous participants perceived individuals with behavioural disorders to be, the greater their desire to maintain social distance [52]. This could suggest that the more negative attitudes towards the hypothetical individual living with BP expressed by the participants in the present study are a result of respondents’ desire to maintain a greater social distance from them due to the belief that their behavior may be unpredictable and, therefore, a threat. In addition, Hong et al. [4] claimed that typically developing children are more likely to be willing to include a child with a disability in a particular activity (such as collaborating to complete a puzzle) when the disability is unlikely to significantly impact the proposed activity; however, when the disability *is likely* to significantly impact the proposed activity (such as when kicking a ball), typically developing children are *less likely* to be willing to include a child with a disability. Additional research is required to determine the precise reasons why respondents’ attitudes and behaviours toward individuals with behavioural disorders are likely to be less positive than their attitudes and behaviours toward individuals with physical disabilities [28].

### Hypothesis 2

The findings did not support H2 (*Out-of-school contact with people with disabilities has a significant positive effect on the attitudes of female*, *non-disabled*, *Saudi elementary school students towards those with disabilities*). Results demonstrated that, in general, facilitating *out-of-school contact* between children with disabilities and non-disabled children has a small positive effect on the attitudes of the latter towards the former (*p* = 0.048; PES = 0.006), this is not the case for specific disabilities, where no significant effect was recorded. In their meta-analysis of 17 studies on inclusive after-school physical exercise programs for children with physical disabilities, Arbour-Nicitopoulos et al. [53] found that participation rates were higher in only two of the studies. Children and young people with physical disabilities and typically developing children and young people reported having diverse interests, suggesting that the peer interactions in certain programs may have been relatively superficial. Some typically developing participants viewed their role in the program as that of "helpers" rather than friends to adolescents living with physical disabilities, whereas the latter were more likely to see these friendships as mutual. In one study, young people with physical disabilities reported feeling a sense of being unwelcome when playing with their typically developing peers [54]. Some researchers [e.g. 53, 54] assert that teachers play a crucial role in fostering mutually beneficial social relationships between non-disabled children and their peers living with physical disabilities: The former are more likely to form friendships and report having had positive interactions with the latter when these interactions were facilitated by instructors with adequate training in providing adapted physical activities [55, 56]. In the present study, however, the out-of-school contact (having a family member or friend with a disability or having played with a person with a disability) was spontaneous and unstructured, and no specific inclusion program was planned, which may explain the small or no effect on participants’ attitudes.

This result concurs with Al-Kandari [57] who investigated the frequency, intensity, and closeness of students’ interactions with people who have disabilities. Positive attitudes about people with disabilities were linked to both knowing someone with a disability and having a family member with a disability. She also discovered that students’ attitudes about people with disabilities improved after even minimal exposure to people with disabilities. Having a friend with a disability has been linked to more positive attitudes toward disability [12, 34, 58], though Freer’s review of the literature notes that this is not always the case [35, 59]. However, the direction of this association is unclear. However, it is not apparent if having a friend with a disability helps children cultivate more positive attitudes or if positive attitudes predict the establishment of friendships. It’s also possible for this to be a two-way street. Not all studies have discovered favourable associations between friendship and attitude [57], and several factors may moderate the link between friendship and students’ views about disability. In addition, Olaleye et al. [58] discovered that, in contrast to boys, girls whose peers had disabilities reported higher levels of positivity. When Petry [60] accounted for the different types of impairments, she found that friendship was no longer connected with improved attitudes toward those with impairments.

### Hypothesis 3

The results did support H3 (*The attitudes of non-disabled female Saudi elementary school students toward their peers with disabilities do not change significantly when the former have frequent in-school contact with the latter*). Non-disabled children who only had in-school contact with peers with disabilities had relatively negative attitudes toward the latter, both in general and in the case of specific disabilities.

The second type of contact examined in this present study occurs at the school or classroom level which we have called "in-school contact." Contrary to most earlier studies, Saudi elementary school girls’ interactions with disabled classmates caused them to feel negative about people with a disability in general, and in particular, towards those with HD, ID, and BP. Several studies on inclusive education [11, 26, 46, 59, 61–63] have shown that attitudes toward students with disabilities in inclusion classes have changed in a big way. Students without disabilities also had good feelings about their friends with ID [35], physical disabilities [37], and HD [64]. Larger inclusive courses were also connected with more positive attitudes toward people with disabilities, as reported by Petry [60]. Schwab [27], however, argued that having students with and without disabilities in the same classroom did not automatically lead to more positive attitudes towards disability, but that encouraging students to voluntarily participate in collaborative activities did. Specifically, Barr and Bracchitta [16] noted that It’s crucial to differentiate between the sheer volume of interactions with people with impairments and the quality of those interactions. McManus et al. [20] affirmed that positive attitudes toward people with disabilities were uniquely predicted by increasing the quality of interaction, while the increased quantity of contact and increased awareness of disabilities were unrelated to positive attitudes. After collaborating with a child who has a disability on a school project, typically developing children’s attitudes toward that child’s friend with a disability improved [65]. Students’ perspectives on inclusive education are influenced by the school climate, as stated by Fu et al. [25]. The norms, goals, values, interpersonal interactions, pedagogical strategies, and administrative framework of a school all contribute to its atmosphere [66]. For the sake of implementing inclusive education, the term "inclusive school climate" is used to describe a relatively consistent and long-lasting environment in which educators and their surrounding community work together [67]. According to McDougall et al. [19], a positive school atmosphere can help children develop positive attitudes toward their peers with disabilities by creating conditions such as fair school goal systems and peer and teacher support [25]. So, it is crucial that we learn more about the contexts in which inclusive education is implemented in Saudi Arabian classrooms. The school’s inclusive strategy, the staff’s part, the impact of campus culture on students unconsciously, and the safety of the school as an institution should all be discussed. According to Fu et al. [25], a principal’s backing is crucial to the expansion of inclusive education in regular schools because it fosters a welcoming environment for all students and helps those with special needs build positive relationships with their teachers and classmates. However, educators also have a responsibility to ensure that their usually developing students have a positive impression of their impaired classmates. Emotional intelligence and self-regulation are also important tools for teachers to use in creating a positive inclusive learning environment [25].

### Hypothesis 4

The results supported H4 (*The attitudes of female Saudi elementary school students toward their peers with disabilities are substantially influenced by the type of school they attend*). To explain, although multiple comparisons showed no significant differences between participants from public, SA, and private schools with regard to attitudes toward disabilities in general, compared with participants from both public and private schools, those from SA schools students expressed more positive attitudes toward the hypothetical individuals with HD and ID yet more negative attitudes toward the hypothetical individual with BP. The conditions mentioned in the above paragraph may have contributed to these differences in Saudi girls’ attitudes toward their disabled peers depending on the type of school they attend. Alhumaid [34] found, contrary to our findings, that participants attending public schools expressed more positive attitudes toward peers with disabilities than those attending private schools when asked about their experiences in PE classes. Different educational strategies and curricula in public and private schools, he suggested, may have a crucial influence in shaping non-disabled students’ perspectives on disability. Private schools in Saudi Arabia are more likely to follow international educational policies and curricula, which may place more of an emphasis on specialized subjects like the sciences, than their public-school counterparts. Alhammad [36], on the other hand, looked at factors that could make it hard to adopt inclusion from the point of view of teachers. He also looked at how students with learning disabilities and regular students are taught in Saudi Arabian elementary schools. He listed inadequate teacher preparation and in-service training, inadequate human support, inadequate resources, inadequate administrative processes, and inadequate infrastructure as some of the factors that could make it hard to implement inclusion in Saudi Arabia.

In the present study, the authors knew that SA schools had different resources and infrastructures than other types of schools. Because of this, they were able to attribute at least some of the differences they saw to the effect of material conditions on how students felt about their disabled classmates. In fact, ARAMCO is a Saudi state-owned company that makes oil, natural gas, petrochemicals, and other products related to oil and natural gas. It is by far the biggest company in the world, with a market value of around $10 trillion in 2015. ARAMCO builds and maintains many public schools for all grades in the Eastern Province of Saudi Arabia. It also gives the schools the tools they need and hires principals and teachers based on certain criteria. So, SA schools are known for having modern, well-equipped buildings and a great learning setting that may influence attitudes toward peers with disabilities [68]. Further research into the things that affect how girls feel about their classmates with disabilities and the differences that may exist between different types of schools in Saudi Arabia will help us figure out how to make sure that all schools have inclusive education.

### Hypothesis 5

Regarding H5 (*Female Saudi elementary school students’ attitudes toward peers with disabilities are positively and significantly determined by their age*), the results indicated that participants’ age had a slight effect on their attitudes towards their peers with HD (*p* = 0.041; Partial Eta Squared = 0.006), while no significant effect of participants’ age was noted on attitudes towards disabilities in general or attitudes towards students with ID or PB. In the literature, results concerning the effect of age on attitude did not give a consistent picture [9]. Some researchers have discovered that older students have more positive attitudes than younger ones, while others have shown the opposite [11, 12, 28]. Another group of researchers [e.g. 69] also found no age-related variations in students’ attitudes toward people with disabilities. More specificity is needed to better understand the attitudes of younger and older students towards disability [26].

These differences may be explained by the fact that each study involved students of different grade levels or ages. Moreover, additional aspects (such as students’ social and emotional development) need to be considered when analysing the correlation between age and students’ attitudes toward disability. In light of this, Babik and Gardner [70] proposed that children’s intergroup prejudice develops in a manner resembling an inverted U: whereas children as young as three have generally favourable attitudes, these attitudes turn increasingly negative between the ages of seven and eight, and then gradually lessen afterward. These authors also argued that the direction in which this overarching chronology can change is context-dependent. It has been said that younger children tend to generalize too broadly and focus on the most obvious aspects of an individual being evaluated, whereas older children can view a situation from many angles and factor in a variety of nuances. In addition, as they become older, children learn to rely less on adults’ instructions and more on their own observations.

Children’s social development in the form of social attributions and group prejudices is also influenced by their knowledge of various disabilities, comprehension of disability, and general cognitive development: Children between the ages of two and seven are in the pre-operational stage of cognitive development; their reasoning is based on perception and symbols; and they tend to focus on the most visible qualities while ignoring less obvious attributes or situational context [71, 72]. Thus, ordinarily developing 5-year-olds prefer to examine just the most visible elements of a challenged peer, such as adapted equipment, while neglecting less visible symptoms connected to the individual’s dyslexia, hyperactivity, intellectual disability, or autism [73, 74]. However, between the ages of 7 and 11, children enter the concrete operational stage of cognitive development; they begin to reason logically, reduce their inclination to overgeneralize, and are better able to examine a situational environment from many perspectives [75, 76]. Typically developing youngsters are more likely to participate in play activities with impaired peers when they have a greater knowledge of impairment [70].

### Study limitations

Several limitations of this study were identified. First, the sample is unlikely to accurately represent the general Saudi elementary-school student population; it was drawn from students living in a single province (the Eastern Province) of Saudi Arabia. As the Eastern Province, known also by “ash-Sharqiyah”, is the largest and one of the most populous provinces in Saudi Arabia, we endeavored to cover a variety of regions within it. Second, due to gender segregation in Saudi education, this research project focused only on all-female elementary schools. It is, therefore, possible that this study’s results cannot be generalized to the Eastern Province’s entire student population, or even that of Saudi Arabia as a whole. Females also appear to be more accepting of people with disabilities than males [48], as demonstrated in adults [18], teenagers [19] and preschool children [10]. However, Alhumaid [34] indicated that in Saudi Arabia, males tend to be more accepting of children with disabilities than women. Future research using representative samples of the Saudi population and including both genders is strongly recommended. Third, the characteristics of each type of contact (quality, quantity, structured, non-structured) were not assessed alongside disability severity, school infrastructure, school services, and the concreteness of inclusion within the school. Other potential confounding variables include the nature of the relationship between people with and without disabilities, integration conditions, and severity of disability. Longitudinal studies on contact and attitude change are needed to determine whether exposure to certain types of disability leads to a more rapid change in perspective than exposure to other types of disability. The final limitation of the study lies in the small effect sizes observed for the various effects of school type and type of contact on attitudes towards students with HD and ID and disabilities more generally. All effect sizes were relatively moderate, with a partial eta squared value of η^2^ = 0.01 indicating a small effect, η^2^ = 0.06 indicating a medium effect, and η^2^ = 0.14 indicating a strong effect [77]. Although the effect size for these variables is small by conventional measures, this is consistent with that observed in the vast majority of studies examining the link between contact and attitudes [16].

### Practical implications

First, those responsible for inclusive education should not be disappointed by the findings of this study; rather, the results call for a thorough re-evaluation of the conditions for inclusion in inclusive institutions, as well as the introduction of new measures. Without clear and explicit support, instructors’ collaborative efforts to promote inclusive practices in education in Saudi Arabia will remain ineffective and unsustainable. Second, it is essential to increase the number of opportunities for individuals with disabilities to interact with the non-disabled in a structured and continuous manner. Leaders could also coordinate a series of extracurricular activities for people with disabilities to increase the opportunities for all non-disabled students to meet and interact with peers with disabilities. Continuing to organize such events will assist the community in accepting and assimilating the inclusion of people with disabilities in all social activities. Third, the results of this study regarding the attitudes of Saudi Arabian students, as well as the results of studies of students from other countries, strongly indicate the importance of providing an inclusive educational environment complete with the necessary adaptations for students with disabilities. Fourth, according to the findings of this study, simply enabling non-disabled students to coexist in a classroom or school with a person/people with a disability does not increase the likelihood of acceptance by the former of the latter. Teachers should organize and promote collaborative activities (particularly enjoyable and competitive activities) to increase the likelihood of positively influencing non-disabled students’ attitudes towards their peers with disabilities, thereby increasing the likelihood that students with disabilities will be made to feel welcome [10]. Lastly, future research should investigate the connection between non-disabled students’ attitudes and their intentions to engage in collaborative activities with peers with disabilities in-school and extracurricularly. In addition, analyzing samples from various Arabic-speaking regions will aid researchers in comprehending the perspectives of non-disabled students and the factors that influence their attitudes toward their peers with disabilities.

## Conclusions

In general, the non-disabled female Saudi elementary school students sampled in this study reported having relatively positive attitudes toward peers with disabilities, specifically toward students with HD, ID, and BP. These findings give us hope that students with disabilities are largely welcomed by their peers in the educational setting and that the process of consolidating educational inclusion in Saudi Arabia is progressing on the right path. However, the findings revealed that the type of disability, type of school, and type of previous contact with peers with disabilities can all have a significant impact on these perceptions in non-disabled students. The female participants’ attitudes toward their (hypothetical) peers with disabilities differed significantly depending on the extent of the disability, with no effect being found in relation to participant age. Attitudes toward students with BP were much less positive, as indicated by a bigger effect size, compared to students with HD or ID. The findings also revealed that students’ attitudes toward their classmates with HD or ID were strongly influenced by their previous interaction with people with disabilities and the type of school they attended. Participants from SA schools had much more positive attitudes toward their (hypothetical) peers with disabilities than those from public schools; those in non-inclusive schools had much more positive attitudes toward their (hypothetical) peers with disabilities than those from inclusive schools. Having had previous contact with people with disabilities influenced the participants’ attitudes toward students with disabilities in general, with no effect being evident due to participant age. Students from non-inclusive schools reported having much more positive attitudes toward their (hypothetical) peers with disabilities than students from inclusive schools; those who had interacted with disabled individuals outside of school had significantly better views than those who had not. A negative correlation was also observed between the attitudes of girls without disabilities toward their peers with BP and the types of schools they attended, demonstrating that students from SA schools expressed the most negative attitudes toward their peers with BP, with no effect being evident for participant age.

## Supporting information

S1 Checklist STROBE statement—checklist of items that should be included in reports of observational studies. (PDF)

S1 Data SPSS raw data. (XLSX)
