# Supplementary material for: Effects of disability type, prior contact, and school setting on attitudes toward peers with disabilities among Saudi female students aged 7 to 12 years
Source: PLoS One. 2023 Sep 8;18(9):e0291274. doi: 10.1371/journal.pone.0291274 (PMC10490877; doi:10.1371/journal.pone.0291274)
Supplement: S1 Checklist — (PDF) [file pone.0291274.s001.pdf]

STROBE Statement—checklist of items that should be included in reports of observational studies

	Item No.	Recommendation	Page No.	Relevant text from manuscript
**Title and abstract**	1	(*a*) Indicate the study's design with a commonly used term in the title or the abstract	1-3	A cross-sectional study
		(*b*) Provide in the abstract an informative and balanced summary of what was done and what was found		Attitudes toward peers with disabilities
**Introduction**				
Background/rationale	2	Explain the scientific background and rationale for the investigation being reported	5	Factors Associated with Children's Attitudes Towards People with Disabilities
Objectives	3	State specific objectives, including any prespecified hypotheses	13	The Present Study
**Methods**				
Study design	4	Present key elements of study design early in the paper	16	Study Design and Setting
Setting	5	Describe the setting, locations, and relevant dates, including periods of recruitment, exposure, follow-up, and data collection	16	Study Design and Setting
Participants	6	(*a*) *Cohort study*—Give the eligibility criteria, and the sources and methods of selection of participants. Describe methods of follow-up<br>*Case-control study*—Give the eligibility criteria, and the sources and methods of case ascertainment and control selection. Give the rationale for the choice of cases and controls<br>*Cross-sectional study*—Give the eligibility criteria, and the sources and methods of selection of participants	18	Participants
		(*b*) *Cohort study*—For matched studies, give matching criteria and number of exposed and unexposed<br>*Case-control study*—For matched studies, give matching criteria and the number of controls per case		
Variables	7	Clearly define all outcomes, exposures, predictors, potential confounders, and effect modifiers. Give diagnostic criteria, if applicable		N/A
Data sources/ measurement	8*	For each variable of interest, give sources of data and details of methods of assessment (measurement). Describe comparability of assessment methods if there is more than one group	19	Research Method Questionnaire
Bias	9	Describe any efforts to address potential sources of bias	21	Reliability and Validity of the Scale
Study size	10	Explain how the study size was arrived at	18	Participants

Continued on next page

Quantitative variables	11	Explain how quantitative variables were handled in the analyses. If applicable, describe which groupings were chosen and why	19	Questionnaire
Statistical methods	12	(*a*) Describe all statistical methods, including those used to control for confounding	22	Data Analysis
		(*b*) Describe any methods used to examine subgroups and interactions	22	Data Analysis
		(*c*) Explain how missing data were addressed	N/A	
		(*d*) *Cohort study*—If applicable, explain how loss to follow-up was addressed<br>*Case-control study*—If applicable, explain how matching of cases and controls was addressed<br>*Cross-sectional study*—If applicable, describe analytical methods taking account of sampling strategy	N/A	
		(*e*) Describe any sensitivity analyses	N/A	
**Results**				
Participants	13*	(a) Report numbers of individuals at each stage of study—eg numbers potentially eligible, examined for eligibility, confirmed eligible, included in the study, completing follow-up, and analysed	23	Demographics
		(b) Give reasons for non-participation at each stage	NA	
		(c) Consider use of a flow diagram	NA	
Descriptive data	14*	(a) Give characteristics of study participants (eg demographic, clinical, social) and information on exposures and potential confounders	22	Demographics
		(b) Indicate number of participants with missing data for each variable of interest	N/A	
		(c) *Cohort study*—Summarise follow-up time (eg, average and total amount)		
Outcome data	15*	*Cohort study*—Report numbers of outcome events or summary measures over time		
		Case-control study—Report numbers in each exposure category, or summary measures of exposure		
		Cross-sectional study—Report numbers of outcome events or summary measures	22	Demographics
Main results	16	(*a*) Give unadjusted estimates and, if applicable, confounder-adjusted estimates and their precision (eg, 95% confidence interval). Make clear which confounders were adjusted for and why they were included		Data were adjusted to the age of the participants (Tables)
		(*b*) Report category boundaries when continuous variables were categorized		N/A
		(*c*) If relevant, consider translating estimates of relative risk into absolute risk for a meaningful time period		N/A

Continued on next page

Other analyses	17	Report other analyses done—eg analyses of subgroups and interactions, and sensitivity analyses	23-25	*Associations between Attitudes toward Peers with Disabilities with the Type of Disability<br>*Attitudes Towards the Three Hypothetical Disabilities
**Discussion**				
Key results	18	Summarise key results with reference to study objectives	26	Discussion
Limitations	19	Discuss limitations of the study, taking into account sources of potential bias or imprecision. Discuss both direction and magnitude of any potential bias	35	STUDY LIMITATIONS
Interpretation	20	Give a cautious overall interpretation of results considering objectives, limitations, multiplicity of analyses, results from similar studies, and other relevant evidence	27-35	Hypothesis 1, 2, 3, 4, and 5
Generalisability	21	Discuss the generalisability (external validity) of the study results	37	PRACTICAL IMPLICATIONS
**Other information**				
Funding	22	Give the source of funding and the role of the funders for the present study and, if applicable, for the original study on which the present article is based	39	ACKNOWLEDGMENTS

*Give information separately for cases and controls in case-control studies and, if applicable, for exposed and unexposed groups in cohort and cross-sectional studies.

Note: An Explanation and Elaboration article discusses each checklist item and gives methodological background and published examples of transparent reporting. The STROBE checklist is best used in conjunction with this article (freely available on the Web sites of PLoS Medicine at http://www.plosmedicine.org/, Annals of Internal Medicine at http://www.annals.org/, and Epidemiology at http://www.epidem.com/). Information on the STROBE Initiative is available at www.strobe-statement.org.
